# Exploring Host Genetic Polymorphisms Involved in SARS-CoV Infection Outcomes: Implications for Personalized Medicine in COVID-19

**DOI:** 10.1155/2020/6901217

**Published:** 2020-10-19

**Authors:** Omar Ramos-Lopez, Lidia Daimiel, Ana Ramírez de Molina, Diego Martínez-Urbistondo, Juan A. Vargas, J. Alfredo Martínez

**Affiliations:** ^1^Faculty of Medicine and Psychology, Autonomous University of Baja California, Tijuana, Baja California, Mexico; ^2^Nutritional Control of the Epigenome Group, IMDEA Food, CEI UAM + CSIC, Madrid, Spain; ^3^Molecular Oncology and Nutritional Genomics of Cancer, IMDEA-Food Institute, CEI UAM + CSIC, Madrid, Spain; ^4^Hospital Universitario HM Sanchinarro, Madrid, Spain; ^5^Servicio de Medicina Interna, Hospital Universitario Puerta de Hierro Majadahonda, Madrid, Spain; ^6^Facultad de Medicina, Departamento de Medicina, Universidad Autónoma de Madrid, Spain; ^7^Department of Nutrition, Food Science, Physiology and Toxicology, Centre for Nutrition Research, University of Navarra, Pamplona, Spain; ^8^Navarra Institute for Health Research (IdiSNA), Pamplona, Spain; ^9^Spanish Biomedical Research Centre in Physiopathology of Obesity and Nutrition (CIBERObn), Spain; ^10^Precision Nutrition and Cardiometabolic Health, Madrid Institute of Advanced Studies (IMDEA-Food Institute), Madrid, Spain

## Abstract

**Objective:**

To systematically explore genetic polymorphisms associated with the clinical outcomes in SARS-CoV infection in humans.

**Methods:**

This comprehensive literature search comprised available English papers published in PubMed/Medline and SCOPUS databases following the PRISMA-P guidelines and PICO/AXIS criteria.

**Results:**

Twenty-nine polymorphisms located in 21 genes were identified as associated with SARS-CoV susceptibility/resistance, disease severity, and clinical outcomes predominantly in Asian populations. Thus, genes implicated in key pathophysiological processes such as the mechanisms related to the entry of the virus into the cell and the antiviral immune/inflammatory responses were identified.

**Conclusions:**

Although caution must be taken, the results of this systematic review suggest that multiple genetic polymorphisms are associated with SARS-CoV infection features by affecting virus pathogenesis and host immune response, which could have important applications for the study and understanding of genetics in SARS-CoV-2/COVID-19 and for personalized translational clinical practice depending on the population studied and associated environments.

## 1. Background

Coronaviruses (CoVs) have become one of the leading pathogens of the latest emerging outbreaks of respiratory disease, representing a serious public health burden worldwide [1]. A novel coronavirus was identified to play a crucial role in the severe acute respiratory syndrome (SARS) in 2003 [2]. Later, the severe acute respiratory syndrome coronavirus-2 (SARS-CoV-2), which produces the disease coronavirus-2019 (COVID-19), has emerged in December 2019. This new virus appears to be highly contagious and has spread rapidly throughout the world, reaching a pandemic state, with important social and health system costs [3].

CoVs are a large family of positive-stranded, single-stranded, enveloped RNA viruses [4]. Relevant pathophysiological mechanisms of CoV, including SARS-CoV and SARS-CoV-2, are related to an exacerbated immunological response that the viral infection produces in the host [5]. In some cases, this reaction is excessive (inflammatory cytokine storm), triggering an extensive tissue damage and body dysfunction [6]. The clinical manifestations of this infection include typical symptoms such as cough, fever, asthenia, and mild respiratory distress. However, more serious respiratory injuries include pneumonia, acute respiratory distress syndrome, and respiratory failure, accompanied by inflammatory outcomes affecting adipokines and other mediators [6]. Furthermore, recent investigations have reported the presence of neurological, renal, hepatic, and cardiac complications in some patients [7]. On the other hand, a group of patients are asymptomatic or with very mild manifestations during the course of the infection, with a relatively short recovery time [8].

Certain risk factors for the chronicity and severity of COVID-19 infection have been reported, including age (over 60 years), male gender, and the presence of concomitant metabolic conditions such as obesity, diabetes, and hypertension [9, 10]. Nevertheless, there is a constant search for biological factors associated with the evolution of COVID-19. Evidence suggests that genetic factors may influence the onset and progression of infectious diseases [11]. In this context, a number of genetic variants, mainly single nucleotide polymorphisms (SNPs), have been associated with the susceptibility/resistance to viral respiratory infections [12]. Until now, the specific role of the genetic make-up in SARS-CoV-2/COVID-19 disease has been insufficiently analyzed, although proinflammatory-related pathways have been involved [6].

Understanding the host genetic factors involved in SARS-CoV infection could contribute to the identification of new therapeutic tools for advancing in the prevention and clinical management of this disease through a personalized medicine approach [13]. Due to the lack of information concerning genetics and SARS-CoV-2/COVID-19 infection, the aim of the present review was to systematically explore the genetic polymorphisms associated with the clinical outcomes in SARS-CoV infection in humans in order to translate this information to SARS-CoV-2/COVID-19 research and clinical implementation.

## 2. Methods

A systematic review was performed in March 2020 to analyze the association of genetic polymorphisms with SARS-CoV infection outcomes. The methodological procedures of this systematic review were performed according to the Preferred Reporting Items for Systematic Reviews and Meta-Analyses Protocols (PRISMA-P) guidelines [14]. Observational (cross-sectional, cohort, or case-control) studies exploring the role of host genetic polymorphisms in SARS-CoV disease (infection susceptibility, disease progression, and clinical outcomes) in adult subjects were included. Articles written only in the English language were selected. Duplicates, reviews, clinical trials, *in vitro* assays, animal experiments, and human studies reporting negative results or focused on other infectious diseases or genetic factors were excluded.

**Figure 1:**
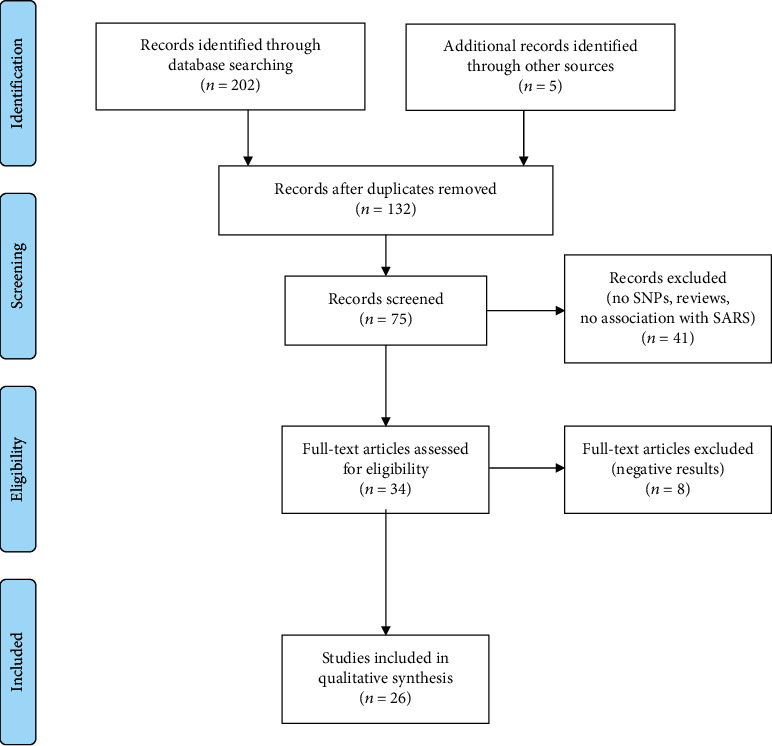
PRISMA flow diagram summarizing the selection of papers included in this systematic review.

The comprehensive literature search encompassed available papers compiled in PubMed/Medline and SCOPUS databases without a period of time or study population restrictions. Advanced search techniques for each database were used to make the search more efficient (i.e., “MeSH” and “tiab” terms). The keywords used were as follows: (“genetic polymorphisms”) OR (“genetic variations”) OR (“gene polymorphisms”) OR (“genetic variations”) AND (“SARS-CoV”) OR (“SARS CoV”) (“SARS coronavirus”) OR (“SARS virus”). The reference sections of the included articles were also scrutinized. This search strategy was replicated at different times to guarantee reproducibility. Moreover, two researchers performed independently the research. Using these terms, a total of 202 articles were identified. Five additional articles were added from reviews, which did not appear in this first stage (Figure 1). First, duplicates were eliminated (*n* = 132), obtaining 75 records for the eligibility criteria assessment. After the duplicates were removed, the titles, abstracts, and keywords of 75 manuscripts were screened, excluding those that did not meet the eligibility criteria (*n* = 41) or reported negative/inconsistent findings (*n* = 8). Finally, 26 articles were included in the current analyses (Figure 1).

The most relevant information about each of the 26 selected studies included in the present review was analyzed using the Population, Intervention, Comparison, Outcome (PICO) criteria [15]. The selected articles met at least 3 inclusion criteria (Supplementary Table 1). The quality assessment of the analyzed studies was performed using the Appraisal tool for Cross-Sectional Studies (AXIS tool), a validated 20-point questionnaire with 6 subdomains (introduction, methods, results, discussion, and others) addressing study quality and reporting encompassing study design, sample size justification, target population, sampling frame, sample selection, measurement of validity/reliability, and overall methods [16]. Because the AXIS tool does not provide a numerical scale for assessing the quality of the study, subjective assessments by the authors are required [16]. Thus, the authors of this study performed a consensus procedure to evaluate the general and overall quality of records in order to select those to be included in the systematic review. The screening was completed with references coming from citations of the 26 selected studies. The results of the AXIS tool are summarized (Supplementary Table 2).

## 3. Results

The present systematic review revealed that 29 polymorphisms located in 21 genes were associated with SARS-CoV susceptibility/resistance, disease severity, and clinical manifestations/outcomes (Table 1).

Available studies used mainly a gene-candidate approach to select those genes involved in disease pathogenesis and phenotypes based on *a priori* knowledge. Thus, genes implicated in key physiological processes such as the mechanisms related to the entry of the virus into the cell and the antiviral immune/inflammatory response were found (Table 1).

Of note, research was predominantly performed in Asian populations, including Chinese, Vietnamese, and Taiwanese populations. Moreover, genetic polymorphisms were mainly SNPs in coding and promoter regions, although insertion/deletion and tandem repeats were also described.

For instance, Chinese individuals homozygous for the tandem repeat domain in exon 4 of the *CLEC4M* gene were less susceptible to SARS infection [17]. Also, the insertion/deletion (I/D) polymorphism in the *ACE1* gene was associated with the progression of pneumonia in SARS patients [18]. Both genes are involved in virus entry.

The rest of the genetic polymorphisms were located in cytokine and chemokine genes implicated in the immune response and inflammatory processes. For example, the *IFN-γ* +874A allele was associated with the susceptibility to develop SARS in two independent populations [19, 20]. Also, the minor alleles of the -88G>T and -123C>A *MxA* promoter SNPs were significantly associated with a lower risk of SARS-CoV infection in Chinese [21]. Nevertheless, the G/T SNP at position −88 in the promoter region of this gene was associated with SARS-CoV disease progression [22].

Genetic variations in the inflammatory *TNF-α* gene were also screened. Thus, whereas the CT genotype at the *TNF-α* -204 locus of this gene was found to be associated with a protective effect on SARS, a higher A allele frequency of the *TNF-α* -308G/A polymorphism was found in the SARS population when compared with healthy controls [23]. Moreover, the prevalence of the *CD14*-159CC polymorphism was significantly higher in patients with severe SARS than in those with mild stages of the disease or controls [24].

The distribution of *HLA* alleles has been widely used as a strategy in the search for the etiology of infectious diseases. In this sense, three genetic polymorphisms in the *HLA-B* gene were related to different SARS-CoV outcomes, including infection predisposition, a protective phenotype [25], and severe forms of the disease [26]. In addition, the Cw∗0801 and Cw∗1502 variants in the *HLA-Cw* gene were associated with SARS risk [27] and SARS resistance [28] in Taiwanese, respectively.

## 4. Discussion

Personalized medicine involves a balanced knowledge of genotype, phenotype, and clinical features of the patient [13], where susceptibility to exacerbated infection is often dependent. Similar to other viral diseases, the first step in SARS-CoV infection includes the attachment of the virus to the host cell receptors [29]. In the present review, genetic variants in *ACE1*, *CLEC4M*, and *CD209* genes were associated with SARS-CoV-related disease pathogenesis. Particularly, the angiotensin-converting enzyme 2 (ACE2, a homologue of ACE1) has been identified as a functional receptor for SARS-CoV [30]. Also, the C-type lectin domain family 4 member M (CLEC4M, also known as L-SIGN) was recognized as a binding receptor for this virus [31]. CD209 (also known as DC-SIGN) shares amino acid identity with CLEC4M, which was involved in facilitating SARS-CoV viral transmission to susceptible cells [32]. These findings demonstrate the important functions of these molecules in the virus entry. Nevertheless, two additional studies failed to demonstrate a relationship between *ACE2* polymorphisms and SARS-CoV outcome in Asians so far [33, 34], emphasizing the need for future research in other populations.

Virus survival and virus-induced disease outcomes (including SARS-CoV) are commonly linked to the modulation of the immune response in the host [35]. As expected, in this review, genetic polymorphisms in genes involved in immunocompetence/inflammatory pathways were found (*ICAM-3*, *IFN-γ*, *RANTES*, *OAS-1*, *MxA*, *IL-12 RB1*, *FcgRIIa*, *MLB*, *CCL2*, *AHSG*, *TNF-α*, *CD14*). Most of them are cytokines or chemokines that are known to be important mediators of early defense against infections, activating protective mechanisms in infected cells [36]. These processes occur during the immune response driven by the SARS-CoV infection and comprise T cell stimulation (*ICAM-3*, *IL-12 RB1*), macrophage activation/deactivation (*IFN-γ*, *AHSG*), cell trafficking (*RANTES*, *CCL2*), RNA degradation and the inhibition of viral replication (*OAS-1*, *MxA*), link between humoral and cell-mediated immune reaction (*FcgRIIa*), virus binding (*MLB*), and inflammation (*TNF-α*, *CD14*) (http://www.genecards.org).

Genetic variations in *HLA* class I or II genes have been often associated with susceptibility/resistance to a wide range of infections, including SARS-CoV [37]. Accordingly, the genes of the *HLA* complex were associated with SARS-CoV outcomes in this study (*HLA-B*, *HLA-DRB1*, *HLA-DRB4*, *HLA-DRB3*, *HLA-Cw*). HLA play a critical role in the presentation of antigenic peptides to T cells, orchestrating an immune response aimed at removing nonself material via neutralization of antibodies, cytokines, and activated cytotoxic T cells [38].

Robust evidence support that genetic variation in human populations contributes to the onset and development of several chronic diseases, including those of an infectious nature [39]. The results of this systematic review show that research exploring the genetic contribution to SARS-CoV infection was predominantly performed in Asian populations, which may be related to its epidemiological origin in China in 2002 [40]. However, allele and genotype frequencies of genetic variants may vary depending on region; therefore, the results should not be extrapolated to other populations without prior exploration. Therefore, further investigation is required to determine the pattern of distribution of these and other candidate polymorphisms in other groups as well as confirm associations with SARS-CoV outcomes. This knowledge is particularly important in populations with heterogenic heritages exposed to absolutely different environmental factors. An example could be Latin American countries, including Mexico, which have an admixture genome with Amerindian, European, and African ancestries; a high prevalence of obesity; and associated comorbidities as well as the adoption of an unhealthy lifestyle (high-fat/sugar diet and physical inactivity) that could exacerbate the outcome of viral infections [41]. Or even some SNPs that have not been associated with SARS-CoV disease outcomes in the Asian population could become a significant association in other populations because of a strong environmental pressure. Thus, we cannot rule out the possibility that other SNPs in other genes could play a role in susceptibility to SARS-CoV infection in these populations.

**Figure 2:**
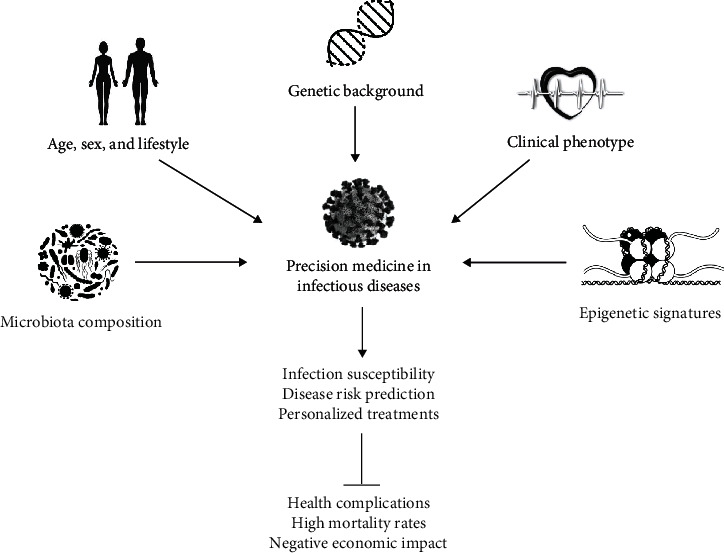
Genetic background and other precision parameters as important predictors of SARS-CoV infection and personalized translational medicine interventions.

In this context, personalized medicine is an integrative therapeutic approach that considers conventional factors (age, gender, clinical phenotype), as well as emerging genetics and interactions with environmental factors to individualize prevention, diagnosis, treatments, and prognosis [42]. Applying the principles of personalized medicine in the current care schemes for the control of infectious diseases (i.e., SARS-CoV) could allow the identification of genetically susceptible groups, disease risk prediction, and personalized therapies to reduce infection-related complications, high mortality rates, and the optimization of economic resources for health care and individualized management of inflammation (Figure 2).

Currently, the high incidence of SARS-CoV-2/COVID-19 around the world, the speed of its spread, and the absence of a specific drug for its pharmacological management emphasize the need to identify risk factors associated with the dynamics of infection and disease progression in order to mitigate its negative impact on the society. A better understanding of the relationship between the genetic make-up and SARS-CoV-2/COVID-19 will provide new insights into the disease pathogenesis by explaining particular phenotypes and clinical responses. Moreover, this knowledge will also aid in identifying biomarkers as potential therapeutic targets for evaluating the efficacy of genome-based interventions and other personalized treatments within the new era of precision medicine.

In addition to the polymorphisms analyzed in this review, new genetic variants affecting SARS-CoV and SARS-CoV-2/COVID-19 susceptibility need to be further explored as well as the interplay of other emerging parameters of individualization such as epigenetic signatures, metabolomic fingerprints, metagenomic data, and lifestyle factors through an integrative holistic approach [43]. Potential gene×drug or gene×inflammation interactions also deserve further investigation. In this regard, drugs targeting the inflammatory cytokines IL-1 and IL-6 have been proposed to reduce extreme immune reaction to the virus and extensive tissue damage [44, 45], where genetics may play a pivotal role. Additionally, genotyping of virus isolates to detect specific multiple mutations is of great importance for the understanding of the evolution and transmission of SARS-CoV infections as well as for vaccine development and disease control [46].

## 5. Conclusion

Although caution must be taken, the results of this systematic review suggest that multiple genetic polymorphisms are associated with SARS-CoV infection outcomes (susceptibility and severity, hospitalization, ICU stays, and medications) by affecting virus pathogenesis and host immune response, which could have important applications for the study and understanding of genetics in SARS-CoV-2/COVID-19 and for personalized translational clinical practice depending on the population studied and associated environments.

## Figures and Tables

**Table 1:** List of relevant polymorphisms in genes implicated in the susceptibility and progression to SARS-CoV infection.

Gene	SNP	Risk/protective allele	Outcome	Frequency	Population	Reference
Mechanisms of entry of the virus into the cell						
*ACE1*	Insertion/deletion (I/D)	D	Higher frequency of hypoxemia	0.33	Vietnamese	[18]
*CD209*	-336A>G	G	Lower blood levels of lactate-dehydrogenase (an independent prognostic indicator for severity of SARS-CoV infection)	0.10	Chinese	[47, 48]
*CLEC4M*	Tandem repeat domain in exon 4	Homozygotes	Lower susceptibility to SARS-CoV infection incidence	0.46	Chinese	[17]
Immune response/inflammation phenomena						
*ICAM-3*	Asp143Gly	Gly	Higher lactate dehydrogenase levels and lower white blood cell counts	0.10-0.17	Chinese	[49, 50]
*IFN-γ*	+874 A/T	A	Higher susceptibility to SARS-CoV infection incidence	0.83	Chinese	[19, 20]
*RANTES*	-28C/G	G	Higher susceptibility to SARS-CoV infection incidence and increased risk of death	0.21	Chinese	[20, 51]
*OAS-1*	A/G SNP in exon 3	G	Higher susceptibility to SARS-CoV infection incidence	0.48	Vietnamese	[22]
*OAS-1*	A/G SNP at the 3'UTR 347 locus of the exon 8	G	Lower susceptibility to SARS-CoV infection incidence	0.24	Chinese	[52]
*MxA*	-123C>A	A	Lower susceptibility to SARS-CoV infection incidence	0.11	Chinese	[21]
*MxA*	G/T SNP at position −88 in the promoter region	G	Higher frequency of hypoxemia	0.76-0.82	Chinese, Vietnamese	[21, 22]
*MxA*	G/T SNP at position −88 in the promoter region	GT genotype	Higher susceptibility to SARS-CoV infection incidence	0.73-0.81	Chinese	[52, 53]
*IL-12 RB1*	+1664 C/T	T	Higher susceptibility to SARS-CoV infection incidence	0.29	Chinese	[54]
*FcgRIIa*	H131R	R	Associated with a severe course of the disease (requiring treatment in an intensive care unit)	0.34	Chinese	[55]
*MLB*	Codon 54 variant (A/B)	B	Higher susceptibility to SARS-CoV infection incidence	0.34-0.35	Chinese	[56, 57]
*CCL2*	G-2518A	G	Higher susceptibility to SARS-CoV infection incidence	0.80	Chinese	[57]
*AHSG*	−799A/T	T	Higher susceptibility to SARS-CoV infection incidence	0.21	Chinese	[58]
*TNF-α*	-204	CT genotype	Lower susceptibility to SARS-CoV infection incidence and increased risk of femoral head necrosis	0.05	Chinese	[23]
*TNF-α*	-308G/A	A	Increased risk of femoral head necrosis	0.06	Chinese	[23]
*CD14*	-159C/T	C	Associated with a severe course of the disease (requiring treatment in an intensive care unit)	0.31	Chinese	[24]
*HLA-B*	B∗0703	B∗0703	Higher susceptibility to SARS-CoV infection incidence	0.11	Chinese	[25]
*HLA-DRB1*	DRB1∗0301	DRB1∗0301	Lower susceptibility to SARS-CoV infection incidence	0.01	Chinese	[25]
*HLA-B*	B∗4601	B∗4601	Associated with severe cases of SARS-CoV infection (deceased or intubated patients)	0.60	Taiwanese	[26]
*HLA-DRB4*	DRB4∗01010101	DRB4∗01010101	Higher susceptibility to SARS-CoV infection incidence	0.51	Chinese	[59]
*HLA-B*	B∗1502	B∗1502	Lower susceptibility to SARS-CoV infection incidence	0.26	Chinese	[59]
*HLA-DRB3*	DRB3∗030101	DRB3∗030101	Lower susceptibility to SARS-CoV infection incidence	0.28	Chinese	[59]
*HLA-DRB1*	DRB1∗1202	DRB1∗1202	Higher susceptibility to SARS-CoV infection incidence	0.47	Vietnamese	[60]
*HLA-Cw*	Cw∗0801	Cw∗0801	Higher susceptibility to SARS-CoV infection incidence	0.23	Taiwanese	[27]
*HLA-Cw*	Cw∗1502	Cw∗1502	Lower susceptibility to SARS-CoV infection incidence	0.10	Taiwanese	[28]
*HLA-DR*	DR∗0301	DR∗0301	Lower susceptibility to SARS-CoV infection incidence	0.11	Taiwanese	[28]

## Data Availability

All data are available in the article.

## Supplementary Materials

Supplementary Materials Supplementary Table 1. PICO criteria used for the inclusion and exclusion of screened studies. Supplementary Table 2. Quality assessment of the individual studies by applying the AXIS tool.
