# Supplementary material for: Exploring Host Genetic Polymorphisms Involved in SARS-CoV Infection Outcomes: Implications for Personalized Medicine in COVID-19
Source: Int J Genomics. 2020 Oct 19;2020:6901217. doi: 10.1155/2020/6901217 (PMC7582067; doi:10.1155/2020/6901217)
Supplement: Supplementary Materials — Supplementary Table 1. PICO criteria used for the inclusion and exclusion of screened studies. Supplementary Table 2. Quality assessment of the individual studies by applying the AXIS tool. [file 6901217.f1.zip › Supplementary Table 1_IJG_2020 (1).docx]

**Supplementary Table 1.** PICO criteria used for the inclusion and exclusion of screened studies.

| **PICO** | **Inclusion criteria** | **Exclusion criteria** |
| --- | --- | --- |
| **Participants** | SARS-CoV positive diagnosis | Participants with other infectious diseases. |
| **Interventions** | Genotyping | Not genotype data |
| **Control/Comparator group** | SARS-CoV negative diagnosis | Not genotype data |
| **Outcome** | SARS-CoV incidence, SARS-CoV-related comorbidities (i.e. hypoxemia, femoral head necrosis) requiring treatment in an intensive care unit, death | Other outcomes not related with SARS-CoV infection |

PICO: Participants, Interventions, Control, Outcomes; SARS: severe acute respiratory syndrome.
