# Supplementary material for: Exploring Host Genetic Polymorphisms Involved in SARS-CoV Infection Outcomes: Implications for Personalized Medicine in COVID-19
Source: Int J Genomics. 2020 Oct 19;2020:6901217. doi: 10.1155/2020/6901217 (PMC7582067; doi:10.1155/2020/6901217)
Supplement: Supplementary Materials — Supplementary Table 1. PICO criteria used for the inclusion and exclusion of screened studies. Supplementary Table 2. Quality assessment of the individual studies by applying the AXIS tool. [file 6901217.f1.zip › Supplementary Table 2_IJG_2020 (1).docx]

**Supplementary Table 2.** Quality assessment of the individual studies by applying the AXIS tool.

| **Assessment Criteria** | **No. of satisfactory studies** |
| --- | --- |
| 1. Were the aims/objectives of the study clear? | 26 |
| 2. Was the study design appropriate for the stated aim(s)? | 26 |
| 3. Was the sample size justified? | 15 |
| 4. Was the target/reference population clearly defined? (Is it clear who the research was about?) | 26 |
| 5. Was the sample frame taken from an appropriate population base so that it closely represented the target/reference population under investigation? | 25 |
| 6. Was the selection process likely to select subjects/participants that were representative of the target/reference population under investigation? | 25 |
| 7. Were the measures undertaken to address and categorise non-responders? | 0 |
| 8. Were the risk factor and outcome variables measured appropriate to the aims of the study? | 24 |
| 9. Were the risk factor and outcome variables measured correctly using instruments/measurements that had been trialled, piloted, or published previously? | 23 |
| 10. Is it clear what was used to determine statistical significance and/or precision estimates? (e.g., P values, Cis) | 18 |
| 11. Were the methods (including statistical methods) sufficiently described to enable them to be repeated? | 25 |
| 12. Were the basic data adequately described? | 25 |
| 13. Does the response rate raise concerns about non-response bias? | 0 |
| 14. If appropriate, was information about non-responders described? | 0 |
| 15. Were the results internally consistent? | 25 |
| 16. Were the results for the analyses described in the methods presented? | 25 |
| 17. Were the authors´ discussions and conclusions justified by the results? | 25 |
| 18. Were the limitations of the study discussed? | 19 |
| 19. Were there any funding sources or conflicts of interest that may affect the authors´ interpretation of the results? | 0 |
| 20. Was ethical approval or consent of participants attained? | 25 |

AXIS: Appraisal Tool for Cross-Sectional Studies
